# Association between the use of information and communication technology and cognitive decline stratified by social isolation: The Otassha study

**DOI:** 10.1016/j.tjpad.2025.100138

**Published:** 2025-03-25

**Authors:** Keigo Imamura, Hisashi Kawai, Manami Ejiri, Hiroyuki Sasai, Kazushige Ihara, Harumi Nakada, Atsushi Araki, Hirohiko Hirano, Yoshinori Fujiwara, Takao Suzuki, Shuichi Obuchi

**Affiliations:** a Tokyo Metropolitan Institute for Geriatrics and Gerontology, Tokyo, Japan; b Hirosaki University School of Medicine, Aomori, Japan; c Komazawa Women's University, Tokyo, Japan; d National Center for Geriatrics and Gerontology, Aichi, Japan

**Keywords:** ICT, Social isolation, Cognitive function, Longitudinal change, Older adults

## Abstract

**Background:**

Prevention of dementia is crucial for reducing its social burden. Social isolation is a known risk factor for dementia. The use of information and communication technology is associated with reduced cognitive decline. However, longitudinal associations of the use of information and communication technology with cognitive function remain unknown, especially for older adults who are socially isolated and at a high risk of cognitive decline.

**Objectives:**

To investigate the association between the use of information and communication technology and changes in cognitive function among older adults with and without social isolation.

**Design:**

Longitudinal observational study

**Setting:**

Data was obtained for two cohorts of community-dwelling older adults aged 65 years with no cognitive impairment (Mini-Mental State Examination score ≥24) at baseline.

**Participants:**

Participants were defined as those who completed baseline assessments of the use of information and communication technology, social isolation, and cognitive function and underwent at least one follow-up assessment of cognitive function in a follow-up survey conducted annually through 2023.

**Measurements:**

The use of information and communication technology was measured using the technology usage sub-items of the Japan Science and Technology Agency Index of Competence. Cognitive function and social isolation were assessed using the Mini-Mental State Examination and the six-item Lubben Social Network Scale, respectively. Data from the two cohorts were combined to examine the association between the use of information and communication technology and changes in cognitive function, as well as the association between the use of information and communication technology and the incidence of cognitive decline (Mini-Mental State Examination <24), using mixed effects models and Cox proportional hazards models, respectively. These analyses were conducted separately based on social isolation.

**Results:**

A total of 1,322 participants (mean age: 72.3 years, 39 % male) were included in the final analysis. The median follow-up period was 3.9 years. Individuals who used information and communication technology experienced a slower rate of cognitive decline than non-users (-0.09, 95 % confidence interval: -0.11 to -0.07 vs. -0.18, 95 % confidence interval: -0.21 to -0.15). In addition, information and communication technology use was associated with a significantly lower risk of cognitive decline (hazard ratio: 0.73, 95 % confidence interval: 0.70–0.76). This association remained consistent among older adults with social isolation (hazard ratio: 0.91, 95 % confidence interval: 0.85–0.97).

**Conclusions:**

The use of information and communication technology was associated with a reduced risk of cognitive decline, even among socially isolated older adults. Creating an environment that enables effective ICT use with appropriate support may help preserve cognitive function in aging populations.

## 1 Introduction

The number of people with dementia is increasing worldwide and is expected to reach 153 million by 2050 [1]. Dementia prevention is important not only to maintain individuals’ quality of life but also to reduce medical and caregiving costs and alleviate the broader societal burden [2]. Social isolation, which indicates an objectively reduced connection with others [3], is thought to increase the risk of dementia; this has been shown by epidemiological data in several studies [4,5]. Increasing social contact in old age may increase an individual's cognitive reserve, encourage healthy behaviors, and reduce the risk of dementia [1]. Therefore, measures to combat social isolation among older adults are an important part of dementia prevention.

With the rapid spread of information and communication technology [ICT] in recent years, many older adults use ICT. In Japan, more than 86 % of the population has a mobile phone or smartphone, and internet use among older people is increasing every year [6]. Previous research suggests that ICT use among older people may prevent cognitive decline [[7], [8], [9], [10]]. For instance, older adults who frequently use computers and the internet tend to experience slower cognitive decline. Furthermore, ICT use can be an intellectually challenging learning activity, especially for older adults who are unfamiliar with new technological devices [7,8]. In addition to reducing the risk of cognitive decline, ICT use has also been shown to alleviate social isolation and promote social interaction. Systematic reviews indicate that ICT use facilitates cognitive, physical, and social activities, including connecting with others, pursuing personal interests, and receiving social support, thereby helping to mitigate social isolation in older adults [11,12].

Although the usefulness of ICT in preventing cognitive decline and reducing social isolation has been reported in several studies, data on its utility in preventing long-term cognitive decline among socially isolated older adults remain limited. Li et al. found that ICT non-users are more likely to experience cognitive decline. However, their study was cross-sectional, and data from longitudinal studies are lacking [13]. Since socially isolated older adults are at higher risk of cognitive decline, clarifying the usefulness of ICT use in this group could provide important information for preventing and addressing long-term cognitive decline among older adults. Therefore, this study aimed to clarify the association between ICT use and longitudinal changes in cognitive function according to social isolation status.

## 2 Methods

### 2.1 Study setting

Data from two independent cohorts in Japan were analyzed. The first cohort was collected from the Otassha Study, an ongoing longitudinal study focusing on comprehensive health checkups among community-dwelling older adults. The Otassha Study began in October 2011 in Itabashi Ward, Tokyo, and involves annual health checkups. At the start of the study, recruitment letters were mailed to all residents aged 65–84 who were registered in the Basic Resident Register, excluding institutionalized residents and participants from previous surveys conducted by our institute. Participants were followed annually, and new participants were recruited each year as they turned 65 years old [14,15]. The second cohort, known as the Toshima Study or the “Toshima Senior Health Investigation of Mind and Activity,” was conducted from 2014 to 2016. Participants aged 65–84 years as of November 1, 2014, who lived in a section of Toshima Ward in Tokyo and were not institutionalized were included [16]. The 2015 survey followed up with participants from the baseline survey. In the 2016 study, in addition to the follow-up, invitations were sent to older adults who did not participate in the baseline venue-invited survey (excluding death or relocation as of 2016). The Itabashi and Toshima Wards, where the two cohort studies were conducted, are urban centers within the 23 wards of Tokyo. This study adhered to the principles of the Declaration of Helsinki.

### 2.2 Analysis cohort

The study included older adults who either participated in the Otassha Study conducted from 2015 to 2019 or in the Toshima Study conducted in 2014 or 2015. The baseline was defined as the first year in which assessments of ICT use, social isolation, and cognitive function were completed. Participants were screened for cognitive function at baseline, and those with low Mini-Mental State Examination [MMSE] scores (<24) were excluded from the analysis. Participants who completed at least one follow-up cognitive function assessment after the baseline year, up to 2023 in the Otassha Study and up to 2016 in the Toshima Study, were included. All participants provided written informed consent, and the study protocol was approved by the Tokyo Metropolitan Institute for Geriatrics and Gerontology (no. R22–034 and no. 32 (2014)).

### 2.3 ICT use

ICT use was assessed using the four-item “Technology usage” scale, a subscale of the Japan Science and Technology Agency Index of Competence, which was developed to assess the higher functional capacity of older adults [17]. This questionnaire has been widely used in studies involving community-dwelling older adults and has also served as an indicator of ICT use in previous research [18]. The four questions defining ICT use were “Can you use a mobile phone?” “Can you use an ATM?” “Can you operate a video recorder such as a Blu-ray recorder or DVD player?” and “Can you send an e-mail by using a mobile phone or computer?” Participants self-reported their responses to each item on a binary scale: “Yes (able)” or “No (unable).” A score of 1 was assigned for “Yes” and 0 for “No.” A higher total score indicated a higher level of ICT usage. A participant who achieved the full score of 4 was categorized as an “ICT user,” whereas any individual with a score of ≤3 was classified as an “ICT non-user.”

### 2.4 Cognitive function

Cognitive function was assessed using the MMSE [19], a cognitive function test with a maximum score of 30 points, consisting of 11 items: time orientation (5 points), place orientation (5 points), immediate and delayed recall of three words (6 points), calculation (5 points), object naming (2 points), sentence repetition (1 point), a three-step verbal command (3 points), written command (1 point), sentence writing (1 point), and figure copying (1 point). Higher MMSE scores indicate higher levels of cognitive function. The MMSE is a structured, orally administered assessment conducted in privacy and was administered to each cohort by well-trained examiners.

### 2.5 Social isolation

Social isolation was assessed using the six-item Lubben Social Network Scale (6LSNS) [20,21]. The 6LSNS has often been used in previous studies as an indicator of social isolation [21]. It evaluates the size of individuals’ active and intimate networks of family and friends with whom they could communicate or whom they could call for help. The 6LSNS consists of a self-rated questionnaire with six items, and each item is rated on a scale of 0 to 5 points. The total score ranges from 0 to 30 points, with high total scores indicating a large social network. Based on previous research, respondents who scored less than 12 points were classified as being socially isolated [21].

### 2.6 Other variables

During the checkups, age, sex, self-rated health, comorbidities (hypertension, stroke, heart disease, diabetes mellitus, hyperlipidemia, osteoporosis, kidney disease, chronic obstructive pulmonary disease, and cancer), instrumental activity of daily living (IADL), living alone, perceived financial status, alcohol consumption, smoking status (current, former, or never), usual gait speed, and years of education were assessed. Self-rated health was rated as follows on a 4-point scale: 1) excellent, 2) good, 3) fair, and 4) poor, with responses 3) and 4) indicating poor [22]. Comorbidities were identified through interviews with pretrained nurses. The participants were categorized according to the number of comorbidities (0, 1, 2, or more). IADL was evaluated with a subscale from the Tokyo Metropolitan Institute of Gerontology Index of Competence [23], where scores range from 0 to 5, with 5 indicating full independence. Perceived financial status was categorized as follows: 1) very comfortable, 2) slightly comfortable, 3) neither comfortable nor hard, 4) slightly hard, and 5) very hard, with responses 4) and 5) classified as hard. Usual gait speed was tested over a 5-m course, with an additional 3 m before and after acceleration and deceleration, timed manually with a stopwatch. Speed was recorded once and calculated by dividing the distance by the time (m/s). The proportion of participants with slow gait speed (<1.0 m/s) was calculated. Years of education were divided into two groups based on whether the participants had received less than 13 years of education.

### 2.7 Statistical analysis

Data from the two cohorts were combined at the individual level. Descriptive statistics are reported as means (standard deviations) and percentages. Unpaired *t*-tests and χ^2^ tests were used to compare baseline characteristics of ICT users and non-users. The baseline characteristics of ICT users and non-users in the socially isolated and non-socially isolated groups were also evaluated.

A multilevel linear mixed-effects model of repeated measures with random intercepts and slopes was used to compare changes in cognitive function between ICT users and non-users because of the multilevel structure of the data, which consisted of individuals nested within two cohorts. The model included the following variables: group (ICT users or ICT non-users), time, an interaction term between group and time, and covariates. To examine the differences in cognitive function changes between ICT users and non-users, interactions between ICT use and time were assessed using the Wald test. The covariates included age, sex, self-rated health, comorbidity categories, alcohol consumption, smoking, IADL, living alone, perceived financial status, slow gait speed, years of education, and baseline MMSE score. Multilevel linear mixed-effects models were applied to the overall cohort, as well as to the socially isolated and non-socially isolated subgroups.

Cox proportional hazards regression analysis was conducted to examine the association between ICT use and future cognitive decline. The incidence of cognitive decline was defined as an MMSE score <24 [24], and the date on which cognitive decline was first noted during the follow-up period was recorded to calculate the follow-up period from baseline. Participants were followed until the first recorded instance of cognitive decline, withdrawal from the study, or study termination (Otassha Study: October 2023; Toshima Study: November 2016). Participants who withdrew from the study were censored at the last recorded assessment date. The covariates were the same as those used in the multilevel linear mixed-effects model. Cox proportional hazards regression analyses were performed for the overall cohort, as well as for the socially isolated and non-socially isolated groups. Cluster effects at the cohort level were considered using robust variance estimates. In addition, to address the potential influence of reverse causation in the Cox proportional hazards regression analysis, a sensitivity analysis was performed using the same adjustment variables after excluding participants who experienced cognitive decline during the first year of follow-up.

For missing data on confounders, multiple imputations were performed using the chained equations method, assuming that the analyzed data were missing at random [25]. The results from 20 imputed datasets were combined for analysis using Rubin's formula. The following variables were incorporated into the imputation model: ICT use, social isolation, cognitive function, covariates, and outcome variables. Statistical analyses were performed using STATA (version 17.0; StataCorp, College Station, Texas, USA). Statistical significance was set at *P* < 0.05.

## 3 Results

### 3.1 Participant characteristics

Of the 1851 study participants from the 2015–2019 Otassha study and the 2014 or 2015 Toshima study, participants with low cognitive function at baseline and those with missing data on ICT use, social isolation, or cognitive function were excluded (*n* = 90). Furthermore, 439 participants for whom follow-up data were missing were excluded; eventually, 1322 participants were included in the final analysis (Supplemental Fig. S1). Table 1 shows the characteristics of the total sample population and the participants divided by ICT use. The mean age of the total sample was 72.3 (6.1) years; 39.0 % of the participants were male, and 29.0 % were socially isolated. The mean baseline MMSE score was 28.8 (1.4). The prevalence of ICT non-users was 34.6 %. Table 2 shows the characteristics of the participants stratified by social isolation and ICT use. In the socially isolated and non-socially isolated groups, the total prevalence of ICT non-users was 49.6 % and 28.5 %, respectively. In the group that experienced social isolation, ICT non-users were characterized by older age, female, slower gait speed, alcohol consumption, smoking status, fewer years of education, and lower baseline MMSE scores compared with ICT users.

**Table 1:** Characteristics of the total study population and participants grouped by ICT use.

		Total	ICT users	ICT non-users	
	Missing	N = 1322	N = 864	N = 458	*P*-value
Age, years	0	72.3 (6.1)	70.7 (5.5)	75.4 (6.0)	<0.01
Male (%)	0	39.0	42.0	33.4	<0.01
Study area	0				<0.01
Otassha study		75.0	78.4	68.6	
Toshima study		25.0	21.6	31.4	
Self-rated health, poor (%)	0	13.7	10.9	19.0	<0.01
Living alone (%)	0	25.5	24.3	27.7	0.17
Self-rated economic status, poor (%)	14	19.5	16.9	24.4	<0.01
IADL, full mark (%)	9	97.1	98.4	94.7	<0.01
Usual gait speed, m/s	3	1.4 (0.3)	1.4 (0.2)	1.3 (0.3)	<0.01
<1.0 m/s (%)	3	6.1	3.5	11.2	<0.01
Comorbidity category (%)	0				<0.01
0		21.7	23.7	17.9	
1		31.8	33.7	28.2	
2+		46.5	42.6	53.9	
Alcohol consumption status (%)	0				<0.01
Current		48.9	54.4	38.4	
Former		6.0	5.0	7.9	
Never		45.2	40.6	53.7	
Smoking status	0				0.02
Current		8.3	9.3	6.3	
Former		26.6	28.1	23.8	
Never		65.1	62.6	70.0	
Years of education <13 (%)	0	53.3	44.4	70.1	<0.01
LSNS total score	0	14.6 (5.9)	15.5 (5.6)	12.9 (5.9)	<0.01
LSNS score <12 (%)	0	29.0	22.3	41.5	<0.01
Baseline MMSE score	0	28.8 (1.4)	29.0 (1.2)	28.4 (1.6)	<0.01

Data are presented as mean (SD) for continuous measures and as % for categorical measures.

IADL: instrumental activity of daily living, ICT: information and communication technology, LSNS: Lubben Social Network Scale, MMSE: Mini-Mental State Examination.

**Table 2:** Characteristics of the participants divided by social isolation and grouped by ICT use.

	No social isolation			Social isolation		
	ICT users	ICT non-users		ICT users	ICT non-users	
	N = 671	N = 268	*P*-value	N = 193	N = 190	*P*-value
Age, years	70.7 (5.4)	75.8 (5.7)	<0.01	70.6 (5.9)	74.9 (6.4)	<0.01
Male (%)	38.0	26.1	<0.01	56.0	43.7	0.02
Study area			<0.01			<0.01
Otassha study	77.1	67.9		82.9	69.5	
Toshima study	23.0	32.1		17.1	30.5	
Self-rated health, poor (%)	9.1	15.7	<0.01	17.1	23.7	0.13
Living alone (%)	22.2	23.5	0.67	31.6	33.7	0.74
Self-rated economic status, poor (%)	14.4	22.6	<0.01	25.5	27.0	0.82
IADL, full mark (%)	98.7	95.5	<0.01	97.4	93.6	0.09
Usual gait speed, m/s	1.5 (0.2)	1.4 (0.2)	<0.01	1.4 (0.3)	1.3 (0.3)	<0.01
<1.0 m/s (%)	2.8	6.4	0.01	5.7	17.9	<0.01
Comorbidity category (%)			<0.01			0.33
0	24.0	16.4		22.8	20.0	
1	34.6	29.9		30.6	25.8	
2+	41.4	53.7		46.6	54.2	
Alcohol consumption status (%)			<0.01			<0.01
Current	53.2	36.6		58.6	41.1	
Former	4.0	6.3		8.3	10.0	
Never	42.8	5.1		33.2	49.0	
Smoking status (%)			0.02			<0.01
Current	8.1	7.5		13.5	4.7	
Former	25.9	17.5		35.8	32.6	
Never	66.0	75.0		50.8	62.6	
Years of education <13 years (%)	45.2	71.6	<0.01	42.0	67.9	<0.01
Baseline MMSE score	29.0 (1.2)	28.5 (1.5)	<0.01	28.8 (1.3)	28.3 (1.8)	<0.01

Data are presented as mean (SD) for continuous measures and as % for categorical measures.

IADL: instrumental activity of daily living, ICT: information and communication technology, LSNS: Lubben Social Network Scale, MMSE: Mini-Mental State Examination.

Missing data include self-rated economic status (*n* = 2), IADL (n=6), and usual gait speed (*n* = 3) for the no social isolation group, whereas for the social isolation group, missing data include self-rated economic status (*n* = 12) and IADL (n=3).

### 3.2 Change in cognitive function between ICT users and non-users

**Fig. 1:**
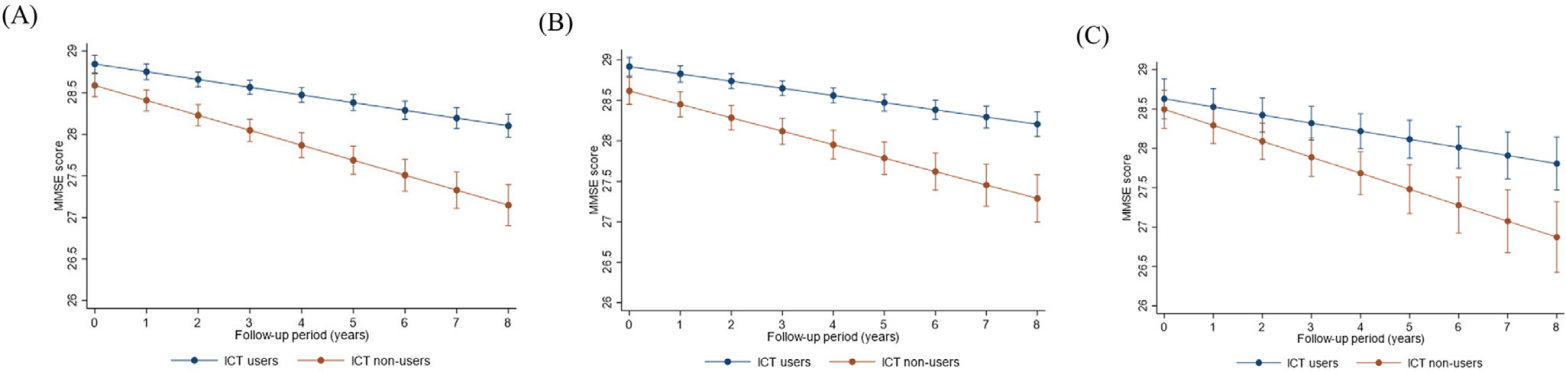
Change in MMSE scores among ICT users and non-users. (A) Total study population, (B) No social isolation group, (C) Social isolation group. The error bars represent 95 % confidence intervals. ICT: information and communication technology, MMSE: Mini-Mental State Examination.

The total number of measurement data points was 6157 (mean: 4.7 times). ICT users showed a significantly slower decline in cognitive function than non-users (Fig. 1: A). The results of the analyses divided by social isolation were also consistent (Fig. 1: B and C). The overall mean annual changes in cognitive function were −0.09 (95 % confidence interval [CI]: −0.11, −0.07) and −0.18 (95 % CI: −0.21, −0.13) for ICT users and non-users, respectively, in the total sample (*P* < 0.01 for group-time interaction; Table 3). In the analyses divided by social isolation, the reduction in ICT users was significantly smaller than that in non-users (*P* = 0.01 for group and time interactions; Table 3).

**Table 3:** Annual change in MMSE scores of ICT users and non-users in the total study population and divided by social isolation.

	Annual change in MMSE score (95 % CI)	*P*-value for interaction
Total study population		<0.01
ICT non-users	−0.18 (−0.21, −0.15)	
ICT users	−0.09 (−0.11, −0.07)	
No social isolation		<0.01
ICT non-users	−0.17 (−0.21, −0.13)	
ICT users	−0.09 (−0.11, −0.06)	
Social isolation		0.01
ICT non-users	−0.20 (−0.26, −0.14)	
ICT users	−0.10 (−0.15, −0.05)	

Interaction term between group (ICT users or ICT non-users) and time.

CI: confidence interval, ICT: information and communication technology, MMSE: Mini-Mental State Examination.

### 3.3 Association between ICT use and incident cognitive decline

During a median follow-up of 3.9 years (interquartile range: 1.9–6.8 years), a total of 123 participants (9.3 %) experienced cognitive decline. Table 4 shows the association between ICT use and cognitive decline. In a model adjusted for participant characteristics, ICT use was associated with a significantly lower risk of cognitive decline (hazard ratio [HR]: 0.73; 95 % CI: 0.70, 0.76). The results were also similar when divided by social isolation status, with a significantly lower risk, particularly in the group with no social isolation.

**Table 4:** Association of ICT use and cognitive decline in the total study population and divided by social isolation.

	Unadjusted model	Adjusted model
	HR (95 % CI)	HR (95 % CI)
Total study population		
ICT non-users	reference	Reference
ICT users	0.37 (0.33–0.41)	0.73 (0.70–0.76)
No social isolation		
ICT non-users	reference	Reference
ICT users	0.35 (0.31–0.39)	0.63 (0.50–0.78)
Social isolation		
ICT non-users	reference	Reference
ICT users	0.50 (0.49–0.51)	0.91 (0.85–0.97)

CI: confidence interval, HR: hazard ratio, IADL: instrumental activity of daily living, ICT: information and communication technology, MMSE: Mini-Mental State Examination. Adjusted for age, sex, self-rated health, comorbidity categories, alcohol consumption and smoking statuses, living alone, self-rated economic status, IADL, years of education, slow gait speed, and baseline MMSE score.

In a sensitivity analysis excluding cases of cognitive decline occurring within the first year, ICT use was associated with a reduced risk of cognitive decline (HR: 0.68; 95 % CI: 0.53, 0.88). These findings were consistent with the results of the main analysis and remained robust when the data were stratified by social isolation (Supplemental Table S1).

## 4 Discussion

In this study, we examined the association between the use of ICT and longitudinal changes in cognitive function. We conducted separate analyses based on the presence or absence of social isolation to investigate whether ICT use serves as a protective factor against cognitive decline among older adults who were socially isolated. Our findings suggest that ICT use is associated with a lower risk of cognitive decline over time. This association was particularly pronounced in older adults who were not socially isolated. In addition, even among those who were socially isolated, ICT use was still associated with a lower risk of cognitive decline.

The finding that ICT use is associated with a lower risk of cognitive decline over time is consistent with previous studies [[7], [8], [9], [10],^26^]. Although the exact mechanisms remain unclear, a plausible explanation for the positive effects of ICT use on cognitive function is that engaging in beneficial intellectual, physical and social activities through ICT use may help maintain and improve cognitive abilities [27,28]. These cognitive activities have been associated with higher episodic memory and lower rates of hippocampal atrophy [29,30]. For older adults unfamiliar with new technologies [12], ICT use can provide cognitively challenging learning activities. In addition, it has been previously shown that the use of multiple devices, as opposed to a single device (such as a desktop or mobile phone), tends to have a stronger protective effect against longitudinal cognitive decline [9]. This suggests that different digital devices may require more frequent cognitive engagement because of their different functionalities and applications [9]. The questionnaire used in this study to assess ICT use included items pertaining to the use of multiple devices, such as mobile phones and computers for e-mail, ATMs, video players, and DVD players. Older adults who engaged in ICT use and were proficient in operating multiple devices may have experienced protective effects against longitudinal cognitive decline.

One of the strengths of this study is that it shows an association between ICT use and longitudinal cognitive decline, stratified by social isolation. Notably, among older adults who were not socially isolated, the combination of existing social connections and cognitively stimulating activities facilitated by ICT use appears to provide a protective effect against cognitive decline [27,28]. Therefore, for older adults who are not socially isolated but do not have adequate access to ICT, integrating ICT into their daily lives with the support of social connections such as family and friends may prevent future cognitive decline.

Another interesting finding of this study was that among older adults who were socially isolated, those who used ICT had a lower risk of cognitive decline than those who did not. The mechanisms underlying the association between social isolation and cognitive decline include fewer activities that stimulate cognitive function [4,31], owing to less interaction with others and less access to health-related information, which reduces the frequency of taking actions that are beneficial for maintaining cognitive function and own health [[32], [33], [34]]. Social interaction influences brain structure, suggesting that it enhances the efficiency of brain network utilization [31]. Previous studies have reported that older adults with less social interaction exhibit reduced overall brain volume and lower gray matter volumes in the temporal lobe, frontal lobe, and other brain regions [35,36]. In addition, social isolation is known to be associated with reduced emotional self-regulation, depression, stress, and anxiety, which have also been shown to be risk factors for cognitive decline and dementia [2,37]. ICT use can help people cope with the limitations associated with aging, such as finding health information and social connections [11,12]. Furthermore, ICT use may afford increased opportunities for diverse experiences that serve as excellent resources and has been reported to contribute to reduced depression in older people [38,39]. Because the study questionnaire did not include a question on the frequency of internet use, the possibility that the ICT users may have greater access to health-related information than the ICT non-user group is speculative. However, the benefits of ICT use may provide a buffer against the negative effects of social isolation on cognitive function.

Although the study results suggest that ICT use is useful in preventing cognitive decline in older adults, many older adults have been found to resist it because of difficulties in adopting new technologies and a limited understanding of how to use them. Previous research has emphasized the need for training and ongoing technical support for older adults regarding ICT use [11,12]. Such educational support has been shown not only to lead to successful ICT adoption [40] but also to have additional benefits for cognitive functioning, such as improved memory and processing speed [[41], [42], [43]]. However, previous research suggests that ICT may help older adults maintain connections with existing social networks, such as their peers, family, and friends, but generally does not contribute markedly to the formation of new social connections [12,44]. Therefore, it is necessary to consider the possibility that ICT use does not fundamentally address social isolation. Therefore, healthcare providers must understand the benefits of ICT use while building support systems to ensure that they are used by older adults.

As reported by the Lancet Commission, social isolation is a known risk factor for dementia in older adults [1]. Recent studies have explored mechanisms linking social isolation to cognitive decline, including alterations in the plasma proteome and changes in hippocampal capacity [45,46]. In contrast, effective intervention strategies for socially isolated older adults have yet to be established, and supporting evidence remains limited [4]. Although the findings of this study do not constitute a direct intervention for social isolation, its strength lies in demonstrating that ICT use is associated with a lower risk of future cognitive decline among socially isolated older adults. This study has some limitations. First, the Japan Science and Technology Agency Index of Competence used to define ICT use includes items such as ATM and video recorder use, which may not necessarily indicate ICT use. Given the annual emergence of new devices and subscriptions, future research should investigate the association between popular contemporary ICT devices and cognitive function. Second, the two cohort studies on which this study is based were conducted in urban areas, where the proportion of ICT users may be higher than that in suburban areas. Therefore, regional differences must be extensively examined in future studies. Third, although this study demonstrated that ICT use is beneficial in preventing cognitive decline, it did not evaluate the optimal frequency and duration of ICT use. Excessive social media use in youth has been reported to negatively affect mental health [47]; thus, future research should explore the potential negative effects of ICT use on health. Fourth, this study did not assess participants' familiarity with ICT devices. Furthermore, it remains unclear whether the ICT use group adopted behaviors beneficial for maintaining cognitive function, such as incorporating mindfulness into their lifestyle during the follow-up period [48,49]. The extent to which these factors influenced longitudinal cognitive function remains unknown. Finally, the study participants were recruited through multiple rounds of invitation-based surveys and did not exhibit cognitive decline at baseline, necessitating caution when generalizing the study results. Future studies should include participants with a wider range of characteristics.

In conclusion, ICT use was associated with a reduced risk of longitudinal cognitive decline and the onset of cognitive impairment. This was also the case for older individuals at a high risk of cognitive decline owing to social isolation. To prevent cognitive decline in old age, it is important to create an environment in which older individuals can effectively utilize ICT with the support of those around them.

## CRediT authorship contribution statement

**Keigo Imamura:** Writing – original draft, Visualization, Software, Resources, Methodology, Investigation, Funding acquisition, Formal analysis, Data curation, Conceptualization. **Hisashi Kawai:** Writing – review & editing, Validation, Supervision, Resources, Methodology, Investigation, Data curation. **Manami Ejiri:** Writing – review & editing, Validation, Supervision, Methodology, Investigation, Data curation. **Hiroyuki Sasai:** Writing – review & editing, Validation, Project administration, Methodology, Investigation, Data curation. **Kazushige Ihara:** Writing – review & editing, Investigation, Data curation. **Harumi Nakada:** Writing – review & editing, Investigation, Data curation. **Atsushi Araki:** Writing – review & editing, Investigation, Data curation. **Hirohiko Hirano:** Writing – review & editing, Methodology, Investigation, Data curation. **Yoshinori Fujiwara:** Writing – review & editing, Methodology, Investigation, Data curation. **Takao Suzuki:** Writing – review & editing, Project administration, Investigation, Data curation. **Shuichi Obuchi:** Writing – review & editing, Resources, Project administration, Methodology, Investigation, Data curation, Conceptualization.

## Declaration of interests

The authors declare the following financial interests/personal relationships which may be considered as potential competing interests:

Keigo Imamura reports article publishing charges and equipment, drugs, or supplies were provided by JSPS KAKENHI. If there are other authors, they declare that they have no known competing financial interests or personal relationships that could have appeared to influence the work reported in this paper.

## Appendix Supplementary materials

Image, application 1

Image, application 2
