# Supplementary material for: Association between the use of information and communication technology and cognitive decline stratified by social isolation: The Otassha study
Source: J Prev Alzheimers Dis. 2025 Mar 25;12(6):100138. doi: 10.1016/j.tjpad.2025.100138 (PMC12434231; doi:10.1016/j.tjpad.2025.100138)
Supplement: Supplementary file 1 [file mmc1.docx]

| Supplemental Table S1. Association of ICT use and cognitive decline in the total study population and divided by social isolation after excluding older adults who experienced cognitive decline during the first 1 year of follow-up (n = 1295) | | |
| --- | --- | --- |
|  | Unadjusted model | Adjusted model |
|  | HR (95% CI) | HR (95% CI) |
| Total study population |  |  |
| ICT non-users | reference | reference |
| ICT users | 0.38 (0.33-0.43) | 0.68 (0.53-0.88) |
| No social isolation |  |  |
| ICT non-users | reference | reference |
| ICT users | 0.37 (0.30-0.45) | 0.57 (0.46-0.71) |
| Social isolation |  |  |
| ICT non-users | reference | reference |
| ICT users | 0.46 (0.40-0.53) | 0.80 (0.67-0.96) |
| CI: confidence interval, HR: hazard ratio, IADL: instrumental activity of daily living, ICT: information and communication technology, MMSE: Mini-Mental State Examination. Adjusted for age, sex, self-rated health, comorbidity categories, alcohol consumption and smoking statuses, living alone, IADL, self-rated economic status, years of education, slow gait speed, and baseline MMSE score. | | |
