# Supplementary figures and images for: Association between the use of information and communication technology and cognitive decline stratified by social isolation: The Otassha study
Source: J Prev Alzheimers Dis. 2025 Mar 25;12(6):100138. doi: 10.1016/j.tjpad.2025.100138 (PMC12434231; doi:10.1016/j.tjpad.2025.100138)

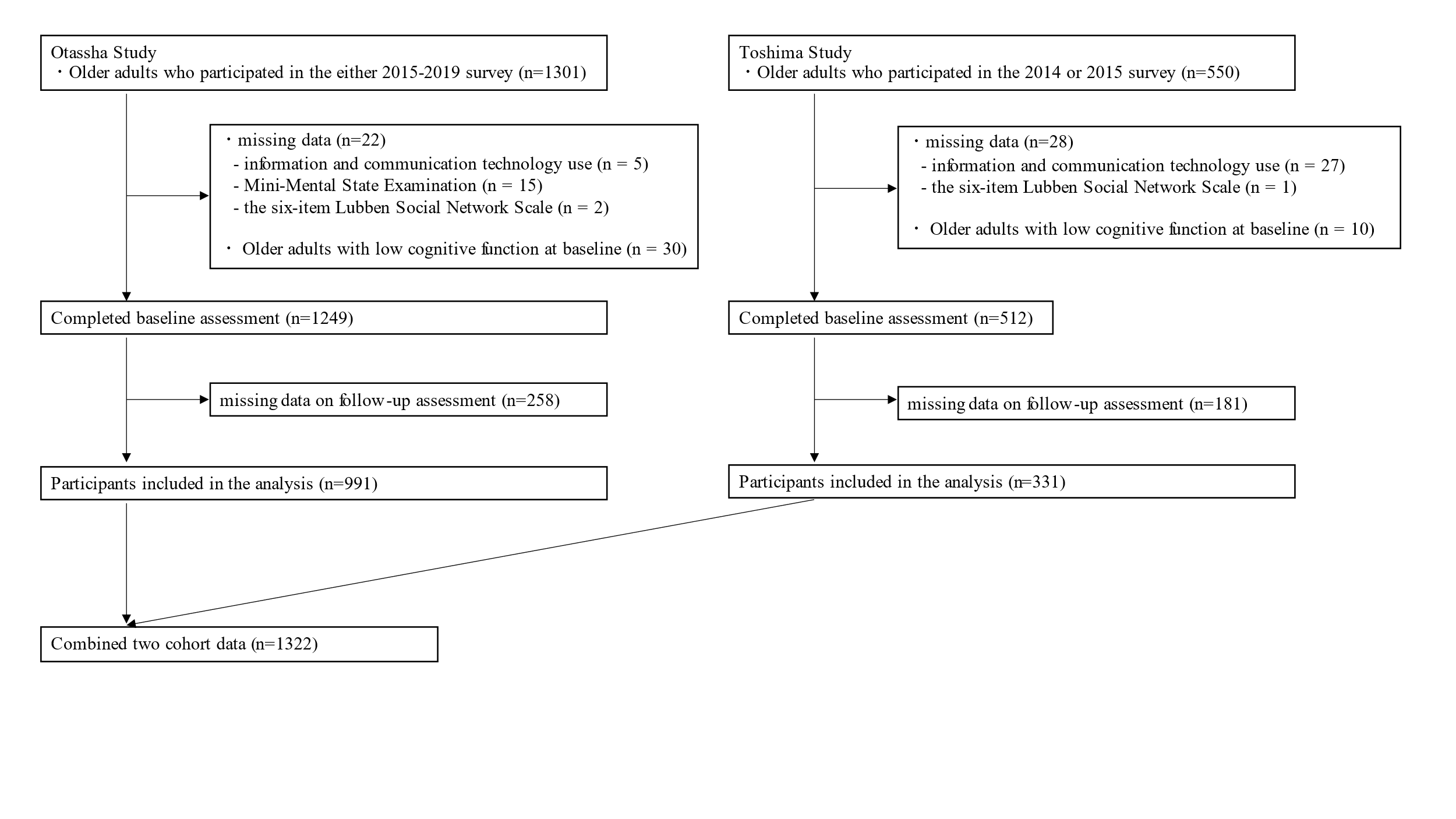


Supplemental Fig. S1 Flow diagram of the participant inclusion and exclusion process

Supplement: Supplementary file 2 [file mmc2.docx]
